# Evaluation of silver nanoparticles synthetic potential of *Couroupita guianensis* Aubl., flower buds extract and their synergistic antibacterial activity

**DOI:** 10.1007/s13205-016-0407-9

**Published:** 2016-03-21

**Authors:** T. Venkata Rajesh Kumar, J. S. R. Murthy, Madamsetti Narayana Rao, Y. Bhargava

**Affiliations:** 1 Department of Botany, Sri Venkateswara University, Tirupati, 517502 India; 2 Department of Biotechnology, Sri Venkateswara University, Tirupati, 517502 India

**Keywords:** *Couroupita guianensis*, Flower buds, Silver nanoparticles, Antibacterial activity, Synergistic activity

## Abstract

The present investigation demonstrates *Couroupita guianensis* flower buds extract mediated synthesis of stable silver nanoparticles (AgNPs). Instant formation of AgNPs was primarily confirmed by the appearance of yellowish brown colour and characteristic silver SPR band in the UV–visible spectrum. Elemental and crystalline natures of the AgNPs were identified from EDX and XRD pattern, respectively. Spherical morphology and the mono-disparity were revealed from TEM and AFM images. The particle size ranged from 5 to 30 nm and average size of 17 nm was consistent in XRD, TEM and AFM measurements. Possible reduction and stabilizing agents, viz., phenolics, flavonoids and proteins were identified from the characteristic FTIR peaks representing their functional groups. The strong antibacterial activity of synthesized AgNPs against Gram-positive and Gram-negative bacteria exhibited the potential for the formulation of synergistic bactericides by combining antibacterial properties of *Couroupita* flower buds extract and silver salts for biomedical applications.

## Introduction

Phytosynthesis methods of silver nanoparticles (AgNPs) are emerging as a potential alternative to physical and chemical processes, ever since the first report on *Geranium* leaf cell free extract mediated synthesis (Shankar et al. 2003), and demonstration of their applications in a number of fields including medicine. The properties and specific applications of AgNPs depend on their shape, size and dispersion. Cell free extract method offers a number of possibilities and flexibility to control various parameters determining the architecture of the nanoparticle. Live plants posses innate nanoparticle synthetic ability as a part of biomineralization, fortification and metal tolerance traits, was evolved to colonize metalliferous soils, this nanoparticle fabrication potential was demonstrated in many plant systems (Marchiol 2012). Efficient and stable synthesis of AgNPs depends on plant species employed and nature of extract.

Varied primary and secondary metabolites of plants, viz., proteins, enzymes, polysaccharides, amino acids and vitamins, antioxidants, flavonoids, flavones, isoflavones, catechins, anthocyanidins, isothiocyanates, carotenoids, polyphenols were attributed as reducing and stabilization agents in the synthesis of metallic nanoparticles (Park et al. 2011). The availability of vast metabolite diversity and unexplored potential of exotic and rare plant systems, a lot of attention is being paid to combine phytochemistry and nanotechnology to develop nanomaterials with desirable size and morphology.

Antimicrobial activity of AgNPs was most extensively evaluated for applications in the medical field to prepare medicines, devices, implants, polymers and dressing material to control infections. Biosynthetic AgNPs are preferred due to their enhanced antimicrobial activity over silver ions and biocompatibility. Antimicrobial activity of AgNPs varies with their size (Panacek et al. 2006), and phytosynthesis of AgNPs with varied size and morphology was reported earlier (Mohanpuria et al. 2008) by employing different plant species.

*Couroupita guianensis,* a member of threatened list (local name: Cannon ball tree) is one of the two species representing *Lecythidaceae* family, found rarely in Botanical gardens and temples in India. Various plant parts viz., leaves, flowers, fruit and bark possess antibacterial, antifungal, antiseptic and analgesic qualities. The flowers are used to cure cold, stomach aches, intestinal gas formation and Diarrhea (Prabhu and Subban 2012).

Generally different parts of flower buds are loaded with antimicrobial and other compounds to protect vital reproductive process. Several chemical constituents with novel structures and bio-active moieties viz. aliphatic hydrocarbon, stigmasterol, alkaloids, phenolics, flavonoids, isatin and terpenoids have been reported from the flowers (Rane et al. 2001; Wong and Tie 1995). Similar phytochemical principles reported from *Couroupita* flowers are implicated in antimicrobial activity, reduction of metal ions and stabilization of AgNPs from other plant systems (Mohanpuria et al. 2008). In the light of the above background, we hypothesize that potential antimicrobial formulations can be developed by combining antimicrobial activity of phytochemicals and their potential to synthesize AgNPs. Here, we report efficient synthesis of AgNPs using *Couroupita* flower bud extract and their synergistic antibacterial activity for the first time.

## Materials and methods

### Preparation of aqueous extract and synthesis of AgNPs

Flower buds [5, 10 and 15 days old] were collected from the plant growing in the Botanical Garden of Sri Venkateswara University, Tirupati. The age of flower bud, concentration and volume of extract of silver nitrate solution for the synthesis of AgNPs were optimized and based on preliminary results, 5 days old, 20 % (FW/Vol.) and 0.01 ml extract/30 ml AgNO_3_ was chosen. The aqueous extract was prepared by boiling 5 g of flower bud pieces in 25 ml of deionized water for 5 min and the filtrate were used as reducing and stabilizing agent. In a typical AgNPs synthesis reaction, 0.01 ml of flower buds broth was added to 30 ml of 10^−3^ M AgNO_3_ and kept in dark at room temperature. The reaction leading to AgNPs synthesis was monitored visually, i.e., the appearance of yellowish brown color and spectral characteristics using UV spectrophotometer.

### Characterization of AgNPs

The reduction of Ag^+^ to Ag^0^ was monitored by UV–visible absorption spectrum (Jasco Corp., UV-530), in the range of 300–700 nm periodically. For the spectral analysis, reaction mixture was diluted with Milli Q water (1:9 ratio) and measurements were carried out as a function of reaction time at room temperature. The colloidal solution of AgNPs was centrifuged for 15 min at 15,000 rpm. This process was repeated for five times by redispersing the pellet into Milli-Q water and the final pellet obtained was air dried and this dried powder of AgNPs was used for further analysis.

A thin film of dried powder of AgNPs on a glass substrate was used to obtain X-ray powder diffraction data by PAN analytical X’Pert PRO diffractometer. The Scanning was done in Bragg–Brentano geometry using step-scan technique and Johansson monochromator to produce pure Cu αK radiation (1.5406 A; 45 kV, 40 MA) in the range of 10° to 90° at a rate of 2 min^−1^. The crystalline size was calculated using the Debye–Scherrer’s equation.

TEM image was acquired and measurements were performed on a JEM-2100 (JEOL, Germany), operated at an acceleration of 300 kV. The Selected Area Electron Diffraction (SAED) pattern was obtained by directing the electron beam perpendicular to one of the spheres.

The thin film on silicon cover glass containing the dried AgNPs sample was analyzed using Atomic Force Microscope (NT-MDT AFM NEXT, Germany). The elemental nature of the nano silver sample was analyzed through EDX using Oxford Inca Penta FET X3 EDX instrument connected to Carl Zeiss EAO MA 15 Scanning Electron Microscope.

FTIR measurements of powdered samples of AgNPs and flower bud extract were carried out on VERTEX K-ALPHA FT-IR Spectrophotometer (Bruker, Germany). Spectra were taken in the wave number range of 500–4000 cm^−1^ without adding the KBr pellets.

### Antibacterial activity

Eight bacterial pathogens, including four Gram positive {(*Bacillus cereus* (MTCC-4079), *Staphylococcus aureus* (MTCC-7443), *Micrococcus luteus* (MTCC-7256) and *Bacillus subtilis* (MTCC-1133)} and four Gram negative strains {*Escherichia coli* (MTCC-1668), *Klebsiella pneumonia* (MTCC-7028), *Pseudomonas aeruginosa* (MTCC-7296) and *Salmonella typhimurium* (MTCC-98)} were obtained from Microbial Type Collection Center, Chandigarh, India for antibacterial studies.

Antibacterial activity of silver nitrate, extract of flower buds and AgNPs was performed following Kirby–Bauer disc diffusion method. Bacterial strains, which are at Log phase (10^8^ cfu/ml) were standardized against McFarland’s standard and were swabbed on to Mueller Hinton Agar (MHA) plates. For the preparation of discs, 20 µl (50 µg/ml) each of the test solutions was used along with standard drug gentamicin (10 µg) for comparison. Cultures were incubated at 37 °C for 24 h and the zone of inhibition was measured by MIC scale. Triplicates were maintained for each treatment. The results were subjected to one-way ANOVA followed by Dennett’s test (**P < 0.05).

## Results and discussion

### Synthesis and UV–visible analysis

Synthesis of AgNPs by reduction of silver ions by the phyto-constituents of the extract were initially observed with the appearance of characteristic yellowish brown color, which is due to excitation of Surface Plasmon Resonance (SPR) phenomena (Mulvaney 1996).

The intensity of color increased, as more and more silver ions got reduced with reaction time and attained stable dark chocolate brown color at 72 h.

**Fig. 1:**
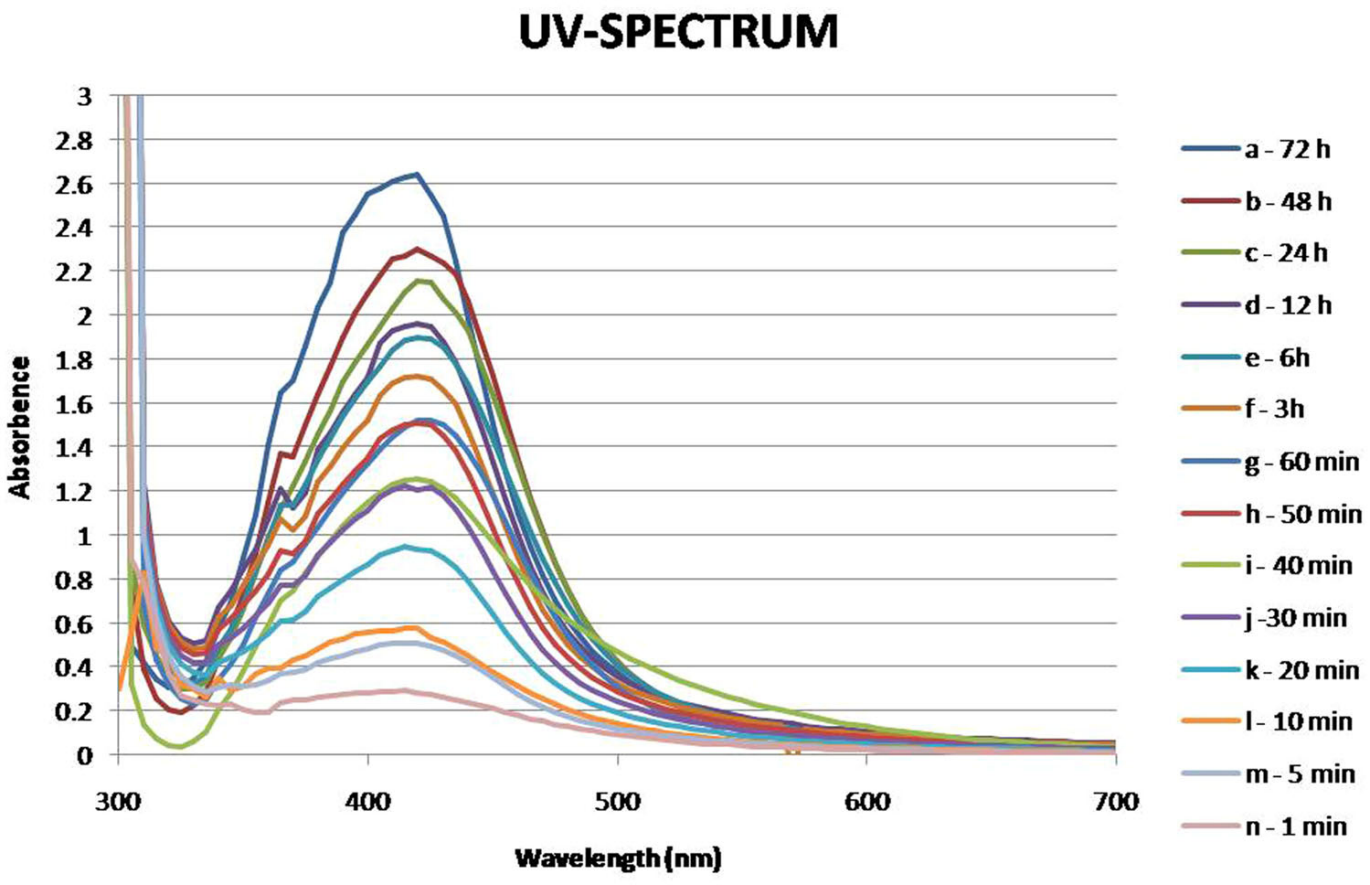
UV–visible analysis of AgNPs showing a typical SPR band from 415–420 nm recorded as a function of reaction time of 10^−3^ M AgNO_3_ aqueous solution with *Couroupita guianensis* flower bud extract

The progress of the silver ion reduction was monitored by UV–vis spectroscopy analysis in the wavelength range from 300 to 700 nm at periodic intervals (1 min to 72 h) (Fig. 1), further synthesis was confirmed by characteristic SPR band at 415–420 nm.

The height of the peak increased with reaction time and was stable at 72 h with an absorption maximum at 420 nm. This absorption maximum is a characteristic feature of AgNPs, as reported from chemical (Kong and Jang 2006), and biosynthesis methods (Logeswari et al. 2012). Generally, the SPR bands are influenced by the size, shape, morphology, composition and the dielectric environment of the prepared nanoparticles (Ahmad et al. 2010). A single, narrow absorption peak centered at 420 nm indicates uniform spherical AgNPs, as expected according to Mie’s theory (Mie 1908).

There was no obvious change in colour intensity, spectral peak position and optical density of the colloid, when monitored at regular intervals over a period of 4 months. This confirms the colloidal stability and uniformity of the hydrosol, as reported earlier in green synthesis studies (Pasupuleti et al. 2013).

The efficiency of plant extract in the synthesis of AgNPs depends on the phytochemical composition and concentration of the extract, the ratio of plant extract to AgNO_3_, time taken for initiation, completion of synthesis and their stability. The extract of 5 days old buds was found to be efficient in terms of the ratio of the extract to silver solution (1:3000), which is several fold less compared to the earlier reports (Usha Rani and Rajasekharreddy 2011) and rate of reduction. Hence, *Couroupita* flower bud extract is more efficient in the reduction of silver ions and stabilization of so formed AgNPs, when compared to the many earlier green synthesis reports (Logeswari et al. 2012). This may be due to the high concentration of reducing agents or more efficient, reducing compounds and stabilizing agents present in the extract at that particular developmental stage of buds.

### Morphological characterization

**Fig. 2:**
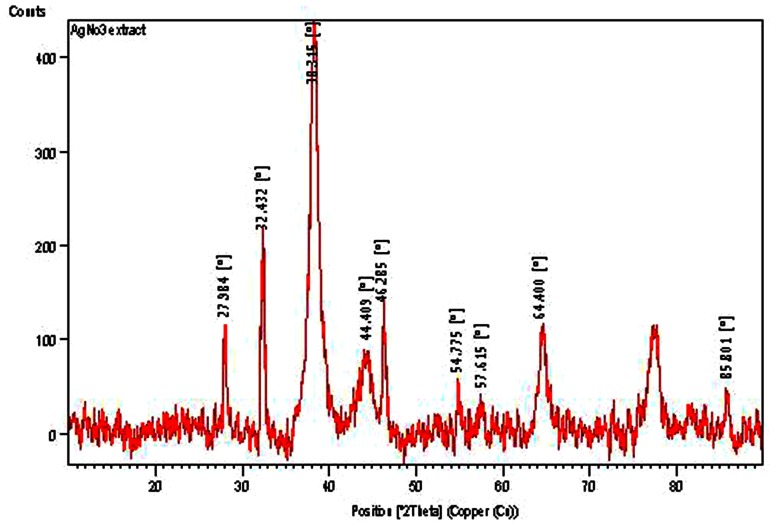
XRD crystallography of phytosynthesized AgNPs showing four prominent peaks of a typical silver crystal

The powder XRD pattern of AgNPs revealed a total of 10 peaks (Fig. 2). The Bragg reflection values of four major peaks 38.314, 44.405, 64.400, and 76.506 at 2*θ* value corresponds to (111), (200), (220) and (311) crystallographic planes respectively, of the face centered cubic structure of a typical silver crystal. These four intense broad peaks reflected a high degree of crystallinity and smaller size of the AgNPs.

The remaining minor peaks are reflections of crystalline organic molecules adsorbed on the surface of the AgNPs. The XRD pattern obtained was consistent with earlier plant based synthesis reports (Murugan et al. 2014). The sizes of AgNPs were determined by estimating the full-width at half maximum (FWHM) of the most prominent peaks from the XRD pattern using the Debye–Scherrer’s equation and the average crystallite size was 17 nm.

**Fig. 3:**
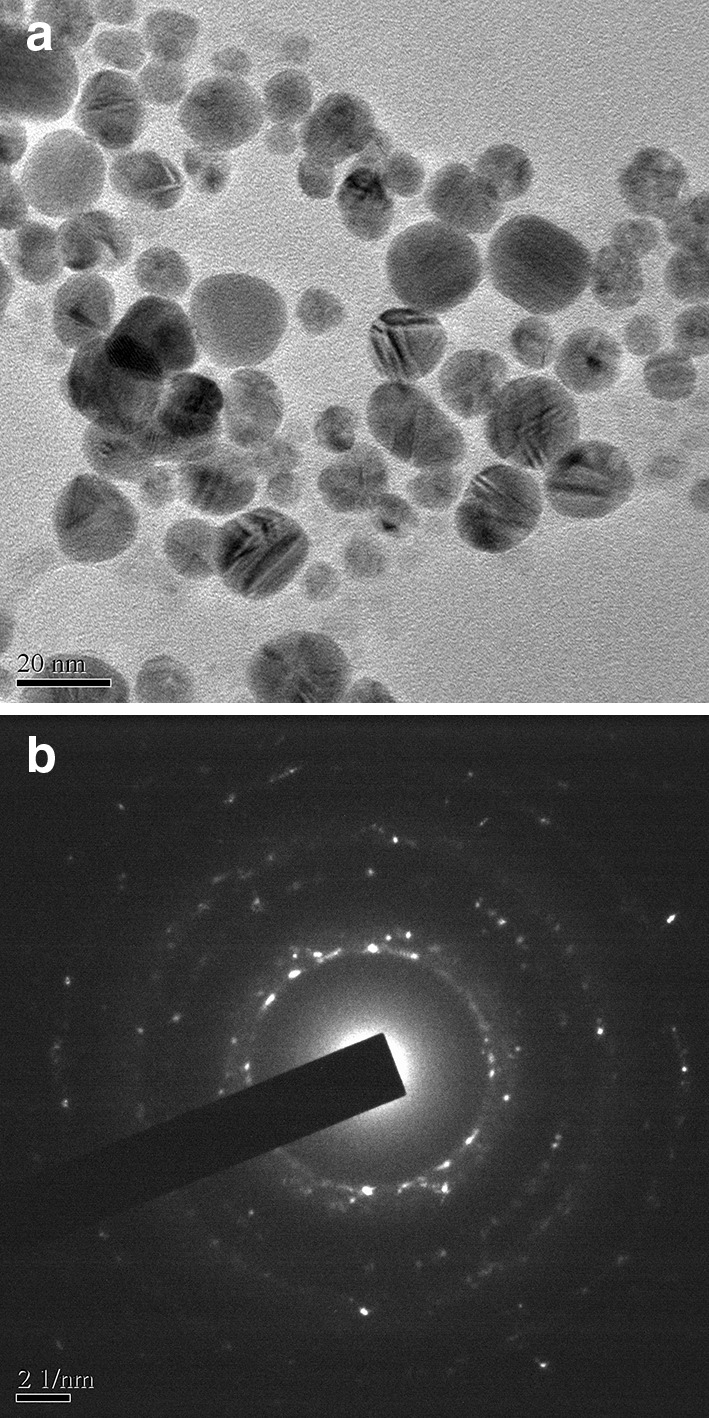
TEM micrograph and SAED pattern of AgNPs. **a** TEM micrograph depicting uniformly dispersed spherical AgNPs. **b** SAED pattern revealing crystalline nature of AgNPs

TEM micrograph showed that the phytosynthesized AgNPs were small, monodisperse and spherical in shape which are in consistent with AFM image. The size of the AgNPs, as can be seen in the image ranged from 5 to 30 nm with an average of approximately 17 nm. These recorded particle size measurements are also in agreement with the estimated values from the AFM and XRD pattern. The SAED pattern (Fig. 3b) revealed distribution and crystalline nature of particles in the focusing zone.

**Fig. 4:**
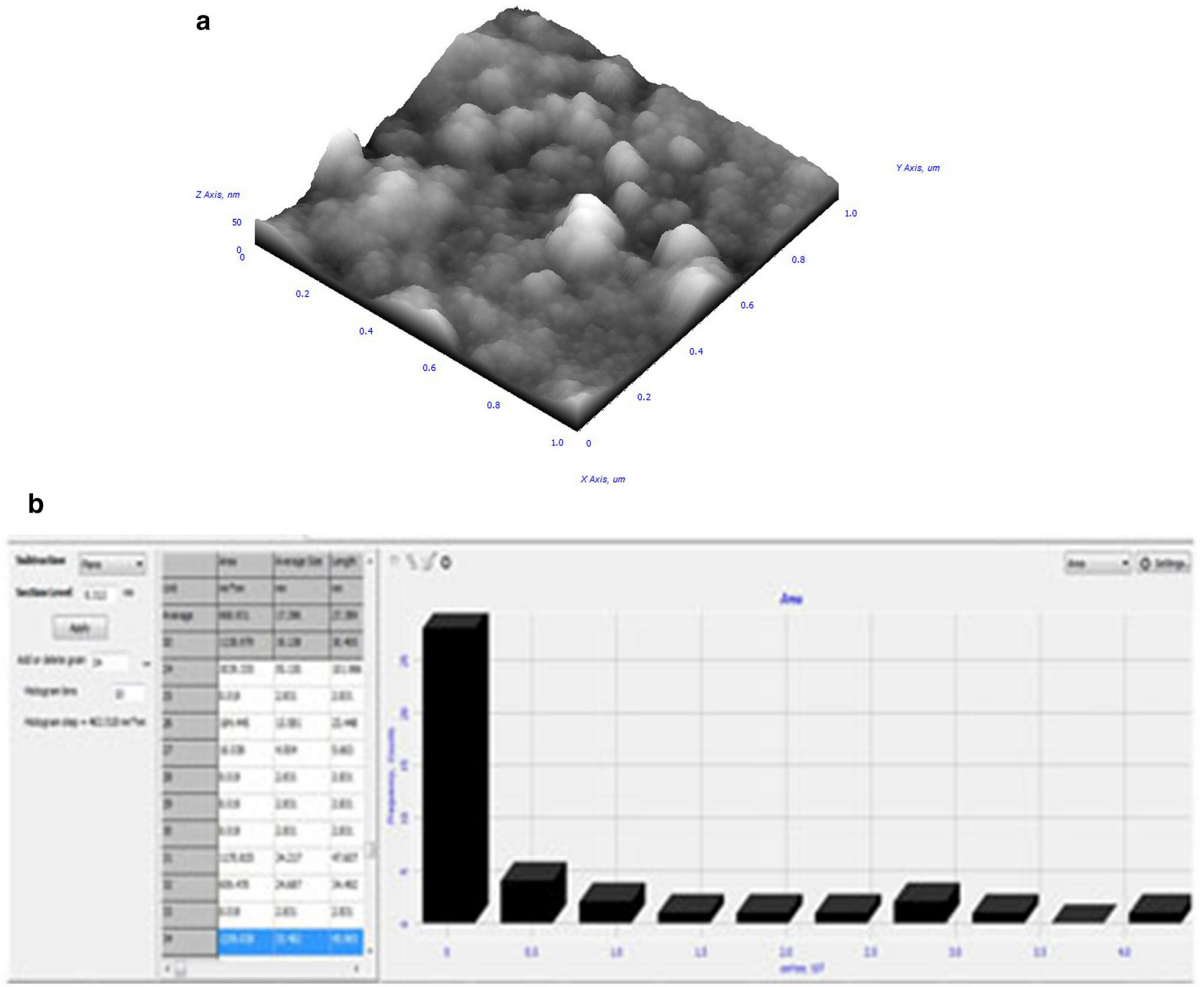
AFM analysis of AgNPs. **a** AFM 3D image depicting spherical shape of AgNPs. **b** Histogram showing the size range of AgNPs

**Fig. 5:**
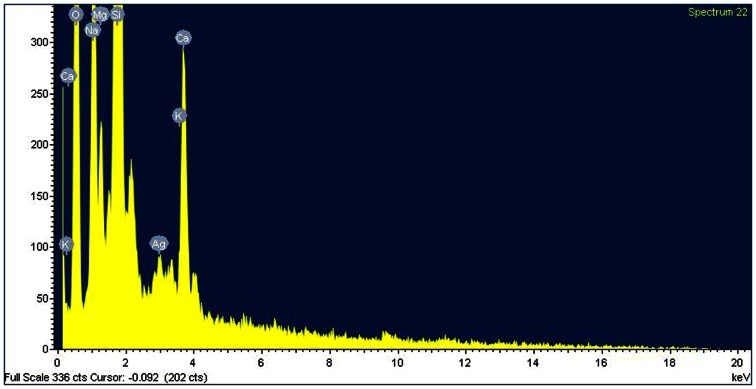
EDX spectrum showing the presence of silver nano crystallite

The surface morphology of synthesized AgNPs can be better visualized and understood by their three dimensional topography. The AFM 3D image (Fig. 4a), of AgNPs depicts uniform spherical morphology and colloidal nature (Fig. 4b). The frequency of particles within the size range of 2.8 to 35 nm was much higher with an average of 17.396 nm, as shown in the particle size histogram (Fig. 4b). The sizes of the AgNPs are in agreement with TEM image and XRD pattern. This size range of particles could be due to capping of AgNPs by various compounds present in the flower bud extract. Previous studies have shown similar variation in size of the AgNPs (Nabikhan et al. 2010). The presence of elemental silver in the colloid was confirmed by the EDX peak pattern in silver region (Fig. 5).

### Phytochemical analysis by FTIR

**Fig. 6:**
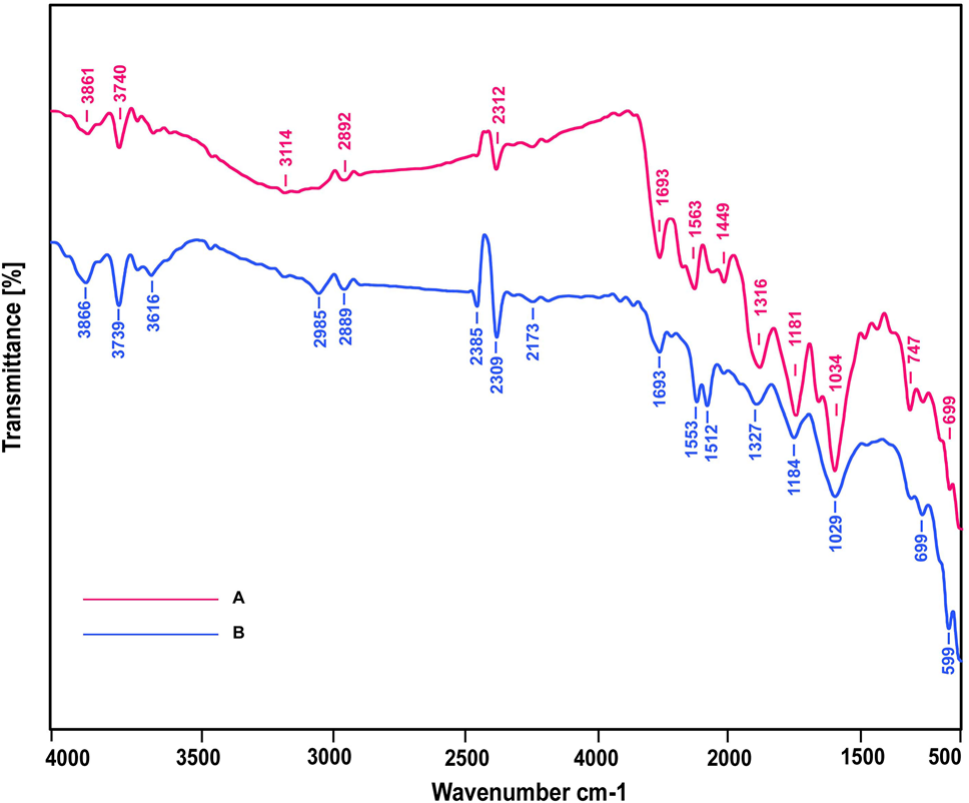
FTIR spectra. *A* Flower bud extract. *B* Stabilized AgNPs showing peaks indicating functional groups of phenolics, aromatic compounds, alcohols and proteins

Metallic nanoparticles generated through phytosynthesis are generally stabilized by phytochemicals through molecular interaction with metal surfaces. The nature of molecular interactions can be studied using FTIR analysis and various capping agents were suggested based on the reference peaks of functional groups in the literature. As shown in (Fig. 6b), there were little changes in the spectrum of AgNPs compared with flower bud extract spectrum (Fig. 6a). The observed peaks in the flower bud extract (Fig. 6a) at 3740 for (O–H) alcohol, 1563 for (N–H) amide, 1181 and 1034 (C=O) for ether, and 2312 for (P–H) phosphine stretches were shifted (Fig. 6b) to lower wave numbers 3739 (OH), 1553 (N–H), 1029 (C=O) and 2309 (P–H) respectively in FTIR spectrum of AgNPs. Whereas, peak at 1316 for (C–N) amine stretch, shifted to higher wave number i.e., 1327 (C–N). There was disappearance of FTIR peak at 1449 for (C–H) alkane and appearance of new peak at 1512 for (N=O) Nitro stretch in FTIR spectrum of AgNPs. Similarity and subtle changes observed in FTIR spectrum of AgNPs compared to the FTIR spectrum of flower bud extract are owing to functional groups characteristic of phenolics, aromatic compounds, alcohols and proteins capping the AgNPs.

Phytochemicals with functional groups representing characteristic FTIR peaks namely phenols, flavonoids, stigmasterol and aliphatic hydrocarbons were reported from *Couroupita guianensis* flowers (Prabhu and Subban 2012; Rane et al. 2001; Wong and Tie 1995). Phenolic compounds are known to have high electron donating property which results in the formation of H radicals, which subsequently reduces silver ions (Ag^+^) to nano size (Ag^0^). The reduction function of polyphenols in the synthesis of AgNPs was also reported earlier (Dibrov et al. 2002). Phenolics posses hydroxyl and carboxyl groups and are able to bind to heavy metals.

The two peaks representing Amide-I are characteristic of proteins, which are responsible for reduction and stabilization of AgNPs, as reported earlier (Gole et al. 2001). The proteins bind to the AgNPs through the free amino group in cysteine residues (Sivaraman et al. 2009). FT-IR spectral characteristics of stabilized AgNPs and different sizes observed in TEM and AFM suggests several phytoconstituents like polyphenolic compounds, flavonoids, and proteins are involved in the synthesis and stabilization of nanoparticles.

### Possible mechanism of AgNPs synthesis

**Fig. 7:**
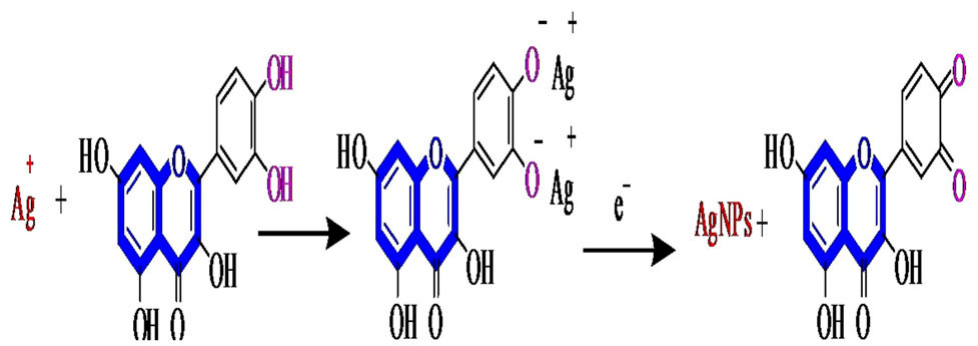
Reduction reaction of silver ions to nanosilver by Quercetin

Quantitative analysis of flowers recorded high amount of quercetin (Prabhu and Subban 2012) and its high reduction potential was also reported (Zhang et al. 2011). It is thus possible that quercetin acts as a reducing agent and it is oxidized by AgNO_3_ resulting in the formation of silver nanoparticles. We therefore propose the overall reaction shown in (Fig. 7), involving reduction of Ag (I) to Ag (0) coupled with catechol oxidation and subsequent cross-linking of the corresponding quinone and quercetin. The redox reaction shown in the reaction scheme indicates the production of two protons per catechol.

### Antibacterial activity of silver nanoparticles

**Fig. 8:**
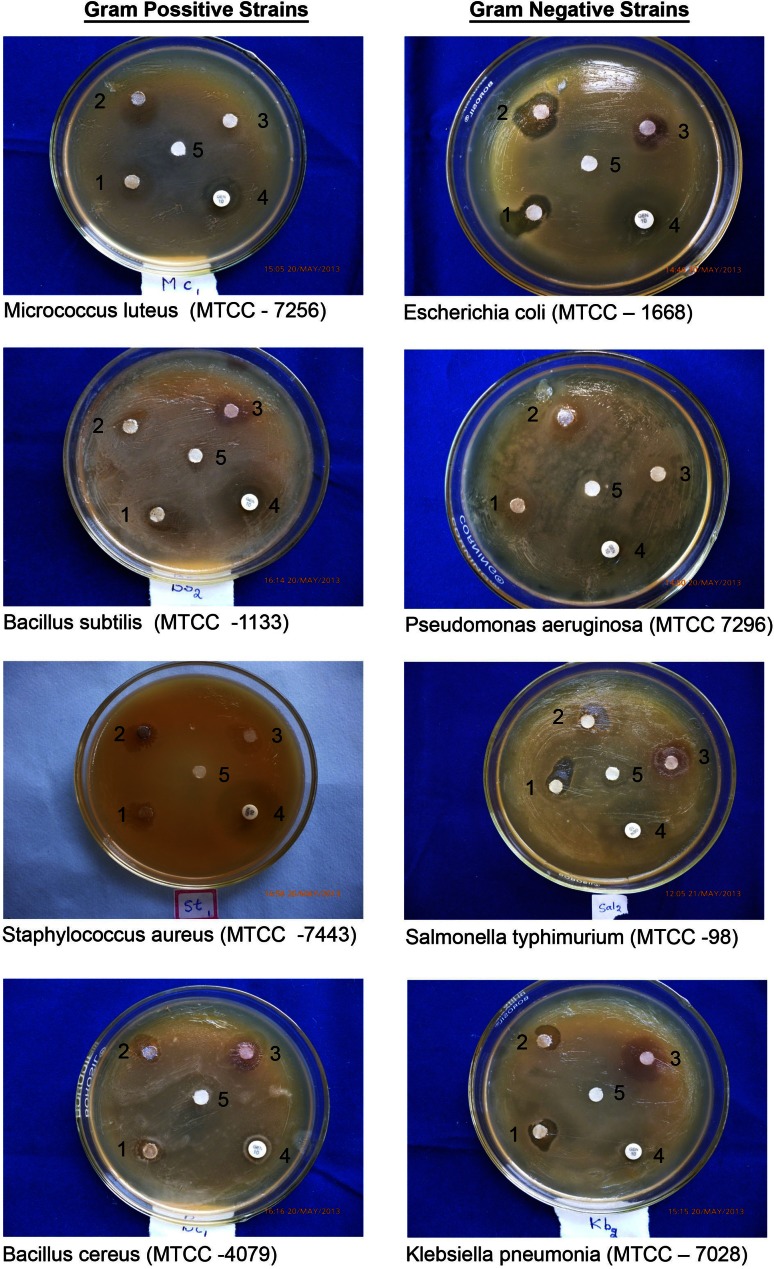
Antibacterial activity of AgNPs on Gram positive and Gram negative strains. *1* AgNO_3_, *2* silver nano solution, *3* flower bud extract, *4* gentamicin, *5* sterile distilled water

The antibacterial activities of AgNPs, AgNO_3_, extract of flower buds and gentamicin against tested Gram positive and Gram negative bacteria were depicted as average values of inhibition zones (Table 1). The degree of sensitivity varied in relation to bacterial species and comparable with standard drug gentamicin under test concentration (Fig. 8).

**Table 1:** Antibacterial activity of AgNO_3_, silver nanoparticles, flower buds extract and gentamicin

Bacterial strains	Zone of inhibition (mm)			
	AgNo_3_ solution	Silver nano solution	Flower bud extract	Gentamicin
Gram +ve				
*Micrococcus luteus* (MTCC-7256)	15 ± 0.1**	24 ± 0.8**	15 ± 0.4**	17 ± 0.6
*Bacillus subtilis* (MTCC-1133)	19 ± 0.3**	23 ± 0.5**	16 ± 0.2**	30 ± 0.4
*Staphylococcus aureus* (MTCC-7443)	16 ± 0.6**	17 ± 0.1**	13 ± 0.1**	18 ± 0.2
*Bacillus cereus* (MTCC-4079)	15 ± 0.7**	20 ± 0.2**	12 ± 0.2**	16 ± 0.1
Gram −ve strains				
*Escherichia coli* (MTCC-1668)	14 ± 0.1**	20 ± 0.6**	13 ± 0.1**	32 ± 0.5
*Pseudomonas aeruginosa* (MTCC 7296)	13 ± 0.5**	18 ± 0.4**	11 ± 0.5**	20 ± 0.3
*Salmonella typhimurium* (MTCC-98)	13 ± 0.2**	17 ± 0.3**	10 ± 0.3**	30 ± 0.4
*Klebsiella pneumonia* (MTCC-7028)	15 ± 0.7**	18 ± 0.1**	13 ± 0.2**	12 ± 0.1

ANOVA followed by Dennett’s test

** P < 0.05

AgNPs showed strong inhibitory activity against four Gram positive bacterial species compared with AgNO_3_ and extract of flower buds. Maximum inhibition zone, i.e. 24 ± 0.8 mm was observed for *Micrococcus luteus* which is closely followed by *Bacillus subtilis* (23 ± 0.5) mm (Table 1 ). Moreover, for *Micrococcus luteus* and *Bacillus cereus*, AgNPs displayed higher bactericidal activity compared to gentamicin and were equally effective for *Staphylococcus aureus*. Notwithstanding, for *B. subtilis*, gentamicin showed significantly higher inhibition than AgNPs. Gram negative bacteria were also significantly inhibited by AgNPs when compared with AgNO_3_ and flower bud extract, while gentamicin displayed strong bactericidal activity against three Gram negative bacteria compared to AgNPs. Only *Klebsiella pneumonia* is less sensitive to gentamicin than AgNPs, AgNO_3_ and flower bud extract.

Even though the antimicrobial activity of silver is well known for over a century, an exact mechanism of action was not yet elucidated. Many possible mechanisms were suggested in the literature that includes, disruption of proton gradient by binding to phospholipids associated with the proton pump of bacterial membranes (Sivaraman et al. 2009), DNA unwinding, inhibition of cell division, damage to bacterial cell envelopes and interaction with hydrogen bonding processes leading to a range of effects from inhibition of growth, loss of infectivity and cell death (Sathish Kumar et al. 2009).

The significant bactericidal activity of the flower bud extract against tested bacterial species is due to the presence of a number of antibacterial compounds like phenols, flavonoids, aromatic compounds, isatin and indirubin (Wong and Tie 1995). Varied mechanisms were suggested in the literature for the antibacterial activity of similar compounds reported from the blossoms of *Couroupita*. Plant phenolic compounds have a range of bioactivities like antibacterial, fungicidal, antiviral, antimutagenic and anti-inflammatory activities (Jayaraman et al. 2010), which are attributed to their disciple hydroxyl (OH) groups. Earlier reports of stigmasterol, aliphatic hydrocarbons and quercetin from flowers (Prabhu and Subban 2012; Rane et al. 2001), also support the observed antimicrobial activity of the extract. Phenolic compounds are known to inhibit enzymes through interaction with sulphahydral groups and proteins nonspecifically (Mason and Wasserman 1987). Quinine, the major plant phenolic compound complexes irreversibly with nucleophilic amino acids present in proteins, leading to inactivation and loss of their function (Stern et al. 1996). The most common targets are surface exposed adhesions, cell wall polypeptides and membrane bound enzymes. Quercitin, belongs to flavonoid class is known to curb *E. coli* gyrase B by binding to the ATP binding site (Plaper et al. 2003), to DNA and induce enzymic DNA damage (Austin et al. 1992). Quercitin also increases permeability of the bacterial inner membrane and loss of membrane potential (Mirzoeva et al. 1997). Multiple modes and overlapping action of these compounds present in the extract were responsible for the antimicrobial activity.

The enhanced antibacterial activity of phyto-chemically stabilized AgNPs is due to the combined effect of nanosilver form and associated antimicrobial plant compounds. This can be explained by the increased surface area to volume ratios in nanoparticles, which provides maximum contact area with bacteria. Hence, bactericidal property of the nanoparticles is size dependent, the larger the surface area, the greater the antibacterial activity (Jeong et al. 2005).

Similar to silver ions, a number of mechanisms were offered in the literature for the disinfectant activity of AgNPs. The proposed mechanisms include, depletion of intracellular ATP through destabilization of the outer membrane and rupture of the plasma membrane (Lok et al. 2006), blocking of respiration by reacting with sulfhydryl (–S–H) groups along the cell wall to form R–S–S–R bonds by the nanosilver causing bacterial cell death (Kumar et al. 2004; Morones et al. 2005). Further, on penetration AgNPs inflict more damage to bacteria by interacting with sulfur- and phosphorous-containing compounds like DNA (Sathish Kumar et al. 2009).

The phytochemicals are reported to have the capability of increasing the susceptibility of bacteria for various drugs (Jayaraman et al. 2010). For example, epigallocatechin gallate enhances the tetracycline action against resistant *Staphylococcus* isolates by impairment of tetracycline efflux pump activity and increased intracellular retention of the drug (Pal et al. 2007). In the present investigation the increased sensitivity of bacteria to the AgNPs is due to overlapping actions of silver and phytochemicals like quercetin, as they bind to the cell membrane, inhibition of enzymes, binding to proteins and DNA resulting in additive mode of action. Damage to the cell membrane by AgNPs was shown by TEM analysis (Pal et al. 2007). The difference in the antimicrobial activity of AgNPs between the Gram positive and Gram negative bacteria is due to the difference in molecular makeup of the cell walls. In the presence of lipopolysaccharide barrier in Gram negative bacteria general susceptibility is very low, as they get protection against toxins and chemicals. Higher sensitivity of Gram negative bacteria to gentamicin than AgNPs may be due to their differential mode of action, as the antibiotic inhibits protein synthesis, while silver failed to disrupt thick cell walls effectively. The difference in the antimicrobial activity of AgNPs among Gram positive and Gram negative bacteria may be due to species/strain variation in uptake, tolerance mechanism and general susceptibility. This is evident from the varying sensitivities of *Bacillus subtilis* and *Bacillus cereus*.

Thus, AgNPs fabricated by phytosynthetic methods enhance the low toxic potential of phytochemicals as antibacterial compounds and also reduce the maximum toxicity of silver on nontargets. Silver and phytochemical synergistic combinations in the form of nanoparticles have potential therapeutic value, as the antibacterial effect is achieved with a lower concentration of silver and phytochemicals. Overlapping and multiple mechanisms of bacteriostatic/bactericidal action of silver and phytochemicals certainly delay the emergence of resistance bacteria.

## Conclusions

The phytochemicals, viz. Phenolic compounds, flavonoids and proteins present in the aqueous extract of flower buds of *Couroupita* exhibited an efficient reduction of silver ions and stabilization of nanoparticles. The average size of spherical nanocrystal was 17 nm and mono-dispersed AgNPs, which are a combination of nano form of silver and phytochemical coating displayed synergism in antimicrobial activity.
